# Development of a protective device for RT-PCR testing SARS-CoV-2 in COVID-19 patients

**DOI:** 10.1017/ice.2020.121

**Published:** 2020-04-13

**Authors:** Tomoya Tsuchida, Shigeki Fujitani, Yukitaka Yamasaki, Hiroyuki Kunishima, Takahide Matsuda

**Affiliations:** 1 Division of General Internal Medicine, St Marianna University School of Medicine, Miyamae, Kawasaki, Japan; 2 Department of Emergency and Critical Care Medicine, St Marianna University School of Medicine, Miyamae, Kawasaki, Japan; 3 Department of Infectious Diseases, St Marianna University School of Medicine, Miyamae, Kawasaki, Japan

*To the Editor*—In December 2019, the novel coronavirus SARS-CoV-2 emerged in Wuhan City, Hubei Province, China, and has now spread worldwide.^1^ Currently, the diagnostic gold standard is the reverse transcription-polymerase chain reaction (RT-PCR). From the sensitivity perspective, sputum samples are preferable for examination.^2^ If acquiring a sputum sample is difficult, a healthcare worker (HCW) can collect sample from the nasopharynx.^3^ Getting a sample from the nasopharynx may carry a risk of the patient sneezing or coughing, and the HCW could be potentially exposed to the virus. Therefore, HCWs are required to wear personal protective equipment (PPE) for each examination and procedure. However, PPE is difficult to don and doff, and donning and doffing carries the risk of infection. Furthermore, if the demand for RT-PCR increases under conditions in which medical resources are scarce, it might be difficult to sample and test all specimens.

**Fig. 1.**
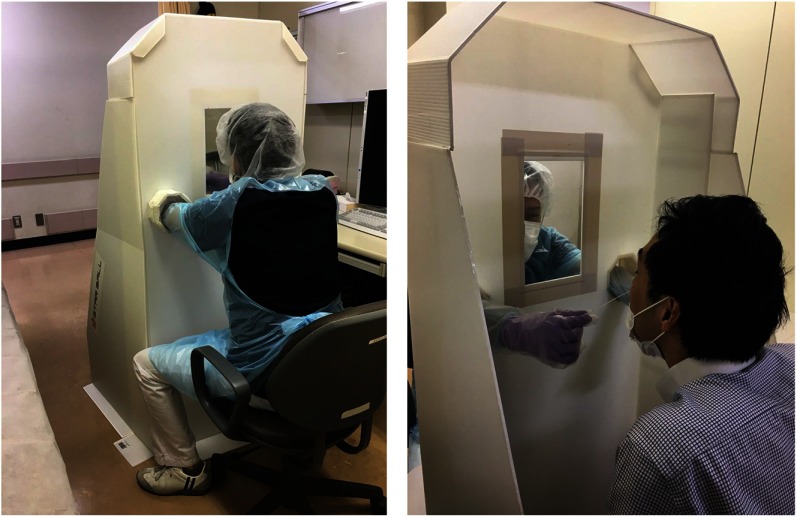
The photo shows the healthcare worker (HCW) and patient sides of the shield. An acrylic plate is used for the window so that the patient’s face can be seen clearly. The HCW wears PPE for examinations but does not need to change PPE for each patient. The disposable gloves used for testing must be changed for each patient.

On March 14, 2020, we developed a protective box (product name, Star Ball Shield) to be used in patients with suspected COVID-19 during clinical examinations or performance of RT-PCR in collaboration with Star Ball Company, Kitakyushu City, Japan (Figure 1). The shield was made by processing waterproof cardboard and is collapsible and easy to carry.

Furthermore, the Star Ball Shield permits HCWs to run RT-PCR without the risk of exposure. This shield liberates the HCWs from the need to don and doff PPE for each clinical examination.

Once a patient’s examination has concluded, the HCW uses alcohol to wipe the surface of the box facing the patient and proceeds to examine the next patient. The Star Ball Shield is extremely helpful in the examination of patients with suspected COVID-19.
